# Equol Attenuates Atherosclerosis in Apolipoprotein E-Deficient Mice by Inhibiting Endoplasmic Reticulum Stress via Activation of Nrf2 in Endothelial Cells

**DOI:** 10.1371/journal.pone.0167020

**Published:** 2016-12-01

**Authors:** Ting Zhang, Qin Hu, Linying Shi, Li Qin, Qianyong Zhang, Mantian Mi

**Affiliations:** Research Center of Nutrition and Food Safety, Institute of Military Preventive Medicine, Third Military Medical University, Chongqing Key Laboratory of Nutrition and Food Safety, Chongqing, P. R.China; University of Basque Country, SPAIN

## Abstract

The development of atherosclerosis is closely related to excessive endoplasmic reticulum stress (ERs). Equol reportedly protects against cardiovascular disease; however, the underlying mechanism for this protection remains unknown. Herein, the mechanisms contributing to the atheroprotective effect of equol were addressed using apolipoprotein E knockout (apoE-/-) mice fed a high-fat diet (HFD) with or without equol. Equol intervention reduced atherosclerotic lesions in the aorta in HFD-fed apoE-/- mice. Plasma lipid analysis showed that equol intervention reduced triglycerides, total cholesterol and LDL-cholesterol and increased HDL-cholesterol. Additionally, equol administration decreased lipid accumulation in the liver. Simultaneously, equol treatment inhibited cell apoptosis induced by *t*-BHP and thapsigargin in human umbilical vein endothelial cells (HUVECs). Furthermore, equol treatment attenuated palmitate, *t*-BHP or thapsigargin-induced upregulation of ER stress markers, including p-PERK, p-eIF2α, GRP78, ATF6 and CHOP proteins expression. The same tendency was also observed in aortic lysates in apoE-/- mice fed with equol plus HFD compared with HFD alone. Moreover, equol treatment dose dependently activated the Nrf2 signaling pathway under oxidative stress. Additionally, elevation of Nrf2 induction was found in aortic lysates in apoE-/- mice fed with a HFD diet containing equol compared with a HFD diet without equol. Importantly, Nrf2 siRNA interference induced CHOP and attenuated the effect of equol to inhibit *t*-BHP mediated CHOP induction, furthermore, abrogated cell apoptosis induced by *t*-BHP, suggesting a role for Nrf2 in the protective effect of equol in HUVECs. Collectively, these findings implicate that the improvement of atherosclerosis by equol through attenuation of ER stress is mediated, at least in part, by activating the Nrf2 signaling pathway.

## Introduction

Cardiovascular disease (CVD) is one of the most prevalent diseases globally and is a major cause of disability and death in humans. Millions of deaths per year are attributed to CVD, and approximately 80% are from developing countries[1]. It is commonly thought that the functional and structural integrity of the endothelium is critical to maintain vascular homeostasis and prevent atherosclerosis[2]. The elevation of free fatty acids such as palmitate increases reactive oxygen species (ROS), which contribute to the development of atherosclerosis in vascular cells[3, 4]. However, the mechanisms by which ROS induce endothelial cell injury remain to be discovered.

Endoplasmic reticulum (ER) stress, a form of intracellular stress that occurs whenever the protein-folding capacity of the ER is overwhelmed, is often implicated in the pathophysiology of various human diseases, including neurodegenerative, cardiovascular and liver diseases[5], diabetic mellitus[6]and obesity[7]. ER stress activates the unfolded protein response (UPR), which involves dissociation of the chaperone BiP/GRP78 from the three ER transmembrane-associated sensor proteins, namely PKR-like ER-regulated kinase (PERK), inositol requiring protein 1α (IRE1α), and activating transcription factor-6 (ATF6), and their subsequent activation[8]. Early activation of the PERK-eIF2α-ATF4-CHOP pathway reduces the protein translation rate to enable the ER to recover from stress; the activation of IRE1-XBP1 and the ATF6-chaperone pathway primarily regulates GRP78, thereby increasing the capacity of protein folding[7, 9]. Therefore, the UPR is essential for the ER to maintain homeostasis from various cellular perturbations. However, persistent ER stress activates a downstream factor of PERK, namely pro-apoptotic gene C/EBP homologous protein (CHOP), resulting in cell apoptosis[10, 11].

Equol (7-hydroxy-3-(49-hydroxyphenyl)-chroman) is a natural specific metabolic product of daidzein[12], which has been reported to have various biological benefits, including cardioprotective effects, anticancer effects, improvement of menopausal syndrome and osteoporosis[13]. Only 30%~50% of the population can produce equol in the gut depending on their particular metabolic phenotype[14]. In contrast to its precursor daidzein, equol has higher antioxidant activity and ERβ affinity[14, 15]. Therefore, it is considered that people who can produce equol benefit more from soy isoflavone than those who cannot perform this endogenous task. The present study implicates that equol ameliorates endothelium function in various types of cells and in vivo models; however, controversy remains, and its mechanisms is poorly understood.

The purpose of the study was to determine the underlying mechanism of the atheroprotective effect of equol. In the present study, apoE-/- mice were fed with a high-fat diet (HFD) containing equol or a HFD diet alone. In addition, human umbilical vein endothelial cells (HUVECs) were challenged with equol in the presence of *t*-BHP or palmitate to examine its role against ER stress and oxidative stress; in particular, the Nrf2 signaling pathway was addressed.

## Materials and methods

### Reagents

Cell culture media HyQ M199/EBSS (M199; SH30351.01) and fetal bovine serum (FBS; SH30370.03) were purchased from HyClone Laboratories (Logan, UT, USA). ECGS (1052) was obtained from SclenCell™ Research Laboratories. *S*-(-)equol for cell culture was obtained from Cayman Chemical Company (Ann Arbor, MI). Equol for in vivo experiments was purchased from Nanjing Enming Pharmaceutical Technology Inc. (Nanjing, China). *tert*-butyl hydroperoxide (*t*-BHP), dimethyl sulfoxide (DMSO; D2650), palmitate (P5585), antibodies against ATF6 (PRS3683) for western blot analysis and thapsigargin were purchased from Sigma-Aldrich (St. Louis, MO, USA). Nrf2 antibody (sc-722), CHOP antibody (GADD153; sc-575), p-PERK antibody (sc-32577), Nrf2 siRNA (sc-37030), and control siRNA (sc-37007) were obtained from Santa Cruz Biotechnology (Santa Cruz, CA). Lipofectamine™2000 transfection reagent (11668–019) was purchased from Invitrogen. NQO1 antibody (A180) was purchased from NOVUS Biologicals (Littleton, CO), activated-caspase-3 p17 (BS7004) was obtained from Bioworld Technology, whereas antibodies against p-eIF2a, eIF2a, and PERK were purchased from Cell Signaling Technology, Inc. The cell counting kit (CCK-8; CK04) was purchased from Dojindo Laboratories (Japan). Total cholesterol (TC) (A110-1), triglycerides (TG) (A111-1), LDL-cholesterol (LDL-C) (A113-1), and HDL-cholesterol (HDL-C) (A112-1) were purchased from Nanjing Jiancheng Bioengineering Institute (Jiangsu, China). The Cell Death Detection ELISA^plus^ kit for the cell apoptosis assay was from Roche (Germany). Oil Red O (O0625) was purchased from Sigma-Aldrich (St. Louis, MO, USA). DCFH-DA was obtained from Beyotime Institute of Biotechnology (Haimen, China). The Annexin V-FITC Apoptosis Detection Kit was purchased from R&D Systems, Inc. (USA).

### Cell culture and treatment

The investigation using Human umbilical vein endothelial cells (HUVECs) conforms with the principles outlines in the Declaration of Helsinki. And the approval was granted by the Third Military Medical University ethics review board. HUVECs were isolated from umbilical cord veins collected from Southwest Hospital of Third Military Medical University (China) as reported previously[16]and cultured on gelatin-coated plastic dishes (Dibco Biocult, Uxbridge, 1–50350) with M199 medium supplemented with 10% heat-inactivated FBS, 2 mM L-glutamine, 30 g/ml ECGS, 20 μg/ml heparin, phenol red, 100 U/ml penicillin, and 100 μg/ml streptomycin. Cells were cultured in a humidified atmosphere in a 5% CO_2_ incubator at 37°C and used at passages 2 to 4 when the cells reached 80~90% confluence. During the logarithmic growth phase, cells were seeded into cell culture plates and cultured overnight to allow cells to attach before further treatment.

### Preparation of palmitate

Initially, two water baths were prepared at 55°C and 70°C. At the same time, 0.4 g of sodium hydroxide was dissolved in 100 ml of distilled water to produce a 0.1 M sodium hydroxide solution; similarly, 1 g of BSA was dissolved in 10 ml of distilled water at 55°C to produce a 10% BSA solution. Next, 26.5 mg of solid palmitate was dissolved in 1 ml of sodium hydroxide solution (0.1 M) at 77°C to acquire 100 mM palmitate. This solution was prepared in duplicate. Finally, 0.4 ml of palmitate (100 mM) was dissolved in 9.6 ml of 10% BSA, and the mixture underwent shaking for 3 h at 55°C to acquire the 4 mM palmitate stock solution. Before use, the palmitate stock solution was filtered and frozen at -20°C.

### Cell viability

Cell viability was assessed using the CCK-8 assay according to the instruction manual. Briefly, HUVECs were seeded in a 96-well microplate (Corning Life Sciences, 3650) at a density of 5,000 cells/well with six replicate wells for each condition on the same plate. After treatment, CCK-8 solution (10 μL/well) was added to the wells, and the plate was incubated at 37°C for 2 h. Sample ODs were measured at 450 nm using a multimode microplate reader (Infinite M200; Tecan, Switzerland).

### ROS

Intracellular ROS was detected using DCFH-DA, an oxidation-sensitive fluorescent probe. After treatment, cells were washed twice in PBS and then incubated with 10 μM DCFH-DA at 37°C for 20 min according to the manufacturer’s instructions. DCF fluorescence intensity was measured using a microplate reader (SpectraMax M2, Molecular Devices) at an excitation wavelength of 488 nm and emission wavelength of 525 nm.

### Flow cytometry

Cells were seeded on 6-well plates; after attachment, cells were treated as needed and then washed twice in 4°C PBS and resuspended in 100 μL of binding buffer at a cell density of 1×10^6^/mL. Cells were then stained with 2 μL of Annexin V-FITC and 2 μL of PI according to the manufacturer’s instructions. They were incubated at 25°C for 15 min in the dark. Samples were acquired on a FACScan flow cytometer (Becton Dickinson) and analyzed using Cellquest software (Becton Dickinson) with 10,000 cells.

### ELISA

The extent of DNA fragmentation within apoptotic cells was determined using the Cell Death Detection ELISA^plus^ Kit (Roche, Germany), which measures cytoplasmic histone-associated DNA fragments using antibodies against biotinylated histone and DNA-POD, according to the manufacturer’s instructions. The absorbance for apoptosis was measured at a wavelength of 440 nm with a reference of 620 nm using a microplate reader.

### siRNA transfection

Nrf2 siRNA transfection was performed according to the manufacturer’s instructions. Cells were seeded in 96- or 6-well culture plates and transfected at approximately 70~80% confluency with the siRNA duplexes using Lipofectamine™ 2000 transfection reagent (11668–019) according to the manufacturer's instructions. The cellular levels of the proteins specific for the siRNA transfection were checked by immunoblotting, and all experiments were performed 24 h after transfection.

### Western blotting

The treated cells were lysed in lysis buffer (50 mM Tris, pH 8.0, 150 mM NaCl, 0.1% SDS, 1% Triton X-100, 0.5% deoxycholate and protease inhibitors), and the protein concentrations of the lysates were measured using the Bradford assay. Briefly, 40~60 μg of protein was resolved by 15% SDS-PAGE and then electroblotted onto polyvinylidene difluoride membranes for western blot analysis. Blots were probed with 1:1,000-diluted primary antibodies overnight at 4°C, followed by the appropriate horseradish peroxidase (HRP)-conjugated secondary antibody (1:10,000 dilution) for 1–2 h. Next, the proteins were visualized by enhanced chemiluminescence exposure to X-ray film. Finally, the blots were scanned, and densitometric analysis was performed on the scanned images using Scion Image-Release Beta 4.02 software.

### Mice and diets

ApoE-/-mice bred onto a C57BL/6 background and purchased from the Jackson Laboratory were housed in micro-isolator cages with filter tops and were maintained on a 12-h-light/-dark cycle in a temperature-controlled room. Sixty male mice (4 weeks old) were randomly assigned to 4 groups (n = 15) and fed with the indicated diets made according to the regular chow formula (except that sesame replaced soybean meal as the protein source) for 12~14 weeks. Mice were fed separately with regular chow (RC), HFD (10% fat and 1% cholesterol), HFD plus a low dose of equol (0.05%), or HFD plus a high dose of equol (0.1%). Mice consumed food and water ad libitum throughout the study period. The study protocol was carried out strictly conforming to the recommendations in the Guide for the Care and Use of Laboratory Animals by the National Institutes of Health and was reviewed and approved by the Institutional Animal Care and Use Committee of the Third Military Medical University (Chongqing, China). Pentobarbital sodium anesthesia (50 mg/kg of body weight) was administered prior to surgical procedures, with maximal effort exerted to minimize suffering.

### Serum lipid profile measurement

After overnight fasting, the eyes of the mice were removed under anesthesia prior to the blood draw. And the measurement of serum lipids including TG, TC, LDL-C and HDL-C concentrations were quantitatively analyzed using the respective commercial kits and were measured at 500 nm using a multimode microplate reader.

### Analysis of atherosclerotic lesion

Atherosclerotic lesions were quantified by en face analysis of the aorta (including aortic arch, thoracic and abdominal regions) and by cross-sectional analysis of the aortic root. Briefly, the whole aorta was separated and dissected free of connective tissues, opened longitudinally and pinned flat on a clean surface. After staining with Oil Red O and sealing with glass, en face images of the aorta were taken, and the surface area occupied by plaques was quantified with Image-Pro Plus software (Media Cybernetics, Bethesda, Md). The lesion area was expressed as a percentage of the total surface area examined. To measure the atherosclerotic lesions at the aortic sinus, the upper sections of hearts were embedded in OCT compound (Sigma-Aldrich, St. Louis, MO, USA) and frozen at −20°C. Sections (10 μm thickness) were collected, beginning at the aortic root and extending for 400 μm. Lesions from 10 alternating sections were stained with Oil Red O and haematoxylin, then were quantified using Optimas Image Analysis software (Bioscan Inc., Edmonds, WA, USA).

### Lipid analysis in the liver

Frozen sections of the liver were stained with Oil Red O solution as described previously. The stained area was then viewed using a microscope at a magnification of 200*. The contents of the hepatic lipid accumulation were quantified by the Image-Pro Plus 6.0.

### Tissue preparation and western blotting

Proteins obtained from separate thoracic and abdominal aortas were separated by SDS-PAGE and blotted onto PVDF membranes. The subsequent steps were similar to those mentioned above.

### Statistical analysis

Quantitative data are expressed as the mean ± SEM values. Statistical significance was analyzed via Tukey’s test and ANOVA using SPSS version 13.0 software (SPSS, Inc.). The difference at either P < 0.05 or P < 0.01 was considered to be statistically significant.

## Results

### Equol ameliorates AS in apoE-/- mice

**Fig 1:**
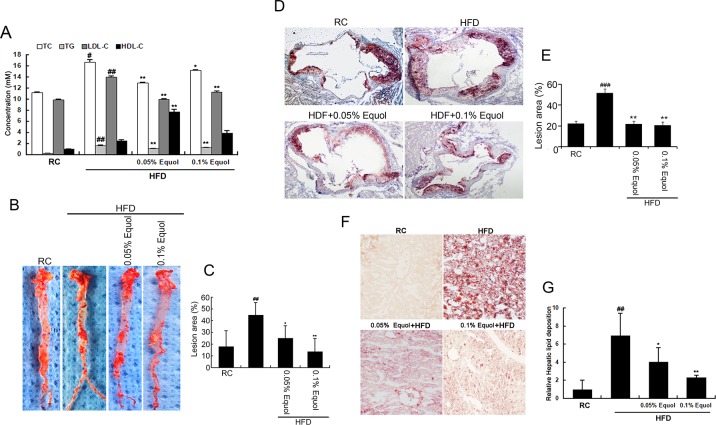
Equol treatment ameliorated atherosclerosis in apoE-/- mice. Mice were divided into four groups: one regular chow group, one HFD group and two HFD groups containing equol (0.05%, 0.1%) for 12~14 weeks. **(A)** After overnight fasting, blood was collected, and the serum was separated. The serum TC, TG, LDL-C, and HDL-C contents were then detected. **(B)** Representative staining of the whole aorta with Oil Red O. En face lesions were quantitatively analyzed using Image-Pro Plus 6.0 (n = 5 per group). **(C)** The bar graphs show the lesion area percentage.**(D)** Representative staining of the aorta sinus with Oil Red O. Lesions were quantitatively analyzed using Optimas Image Analysis software (n = 5 per group). **(E)** The bar graphs show the lesion area percentage. **(F)** The frozen sections of the liver were stained with Oil Red O and viewed under a microscope at 200× magnification. The content of the hepatic lipid deposition was quantified using Image-Pro plus 6.0. **(G)** The bar graphs show the relative contents of hepatic lipid deposition versus the control group. Values are presented as means ± SD. n = 5, ^#^*p* < 0.01, ^##^*p* < 0.001 versus the RC control group; *p < 0.05, **p < 0.01 versus the HFD group.

The apoE-/-mice were weighed at the end of the experiment, and the weight did not differ between groups (data not shown). Compared with HFD, 0.1% equol treatment markedly decreased the concentration of TC, TG, LDL-C (Fig 1A). However, 0.05% equol treatment notably increased the level of HDL-C (Fig 1A).

Staining aortas with Oil Red O demonstrated grossly visible atherosclerotic lesions (Fig 1B, 1C, 1D and 1E). A notable increase in the size of plaques was observed in the HFD groups compared with the RC group, and this increase was markedly attenuated by the administration of equol (0.05% and 0.1%). Simultaneously, equol intervention attenuated the accumulation of hepatic lipid (Fig 1F and 1G). These results suggested that equol improved AS development in HFD-fed apoE-/- mice.

### Equol protects HUVECs from *t*-BHP-induced cell injury

**Fig 2:**
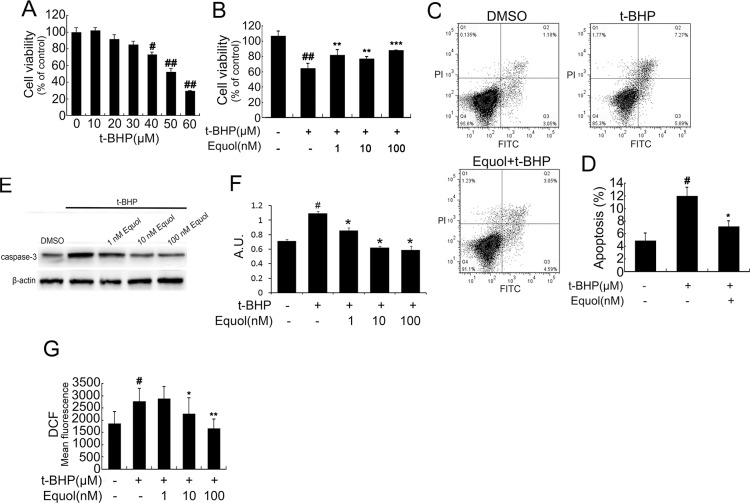
Equol attenuated *t*-BHP-induced apoptosis in HUVECs. **(A)** Cells were treated with different concentrations (0, 10, 20, 30, 40, 50, and 60 μM) of *t*-BHP for 6 h. **(B)** Cells were pretreated with different concentrations (1, 10, and 100 nM) of equol for 24 h followed by treatment with *t*-BHP (50 μM) for an additional 6 h. The control group was treated with 0.2% DMSO. Subsequently, cell viability was measured using a CCK-8 detection kit. **(C)** Cells were treated with or without equol (100 nM) in the presence of *t*-BHP (50 μM). Cell apoptosis was measured by flow cytometry using Annexin V-FITC and propidium iodide (PI) staining. **(D)** The bar graphs show the quantification of the apoptosis percentage. **(E)** Cells were incubated as described in (B), then caspase-3 was determined by western blot. **(F)** The bar graphs show the quantification of the caspase-3 proteins. **(G)** Cells were incubated as described in (B). Next, intracellular ROS was detected by DCFH-DA. Values are presented as means ± SD. n = 3, ^#^*p* < 0.01, ^##^*p* < 0.001 versus the control group; *p < 0.05, **p < 0.01, ***p < 0.001 versus the *t*-BHP-treated group.

**Fig 3:**
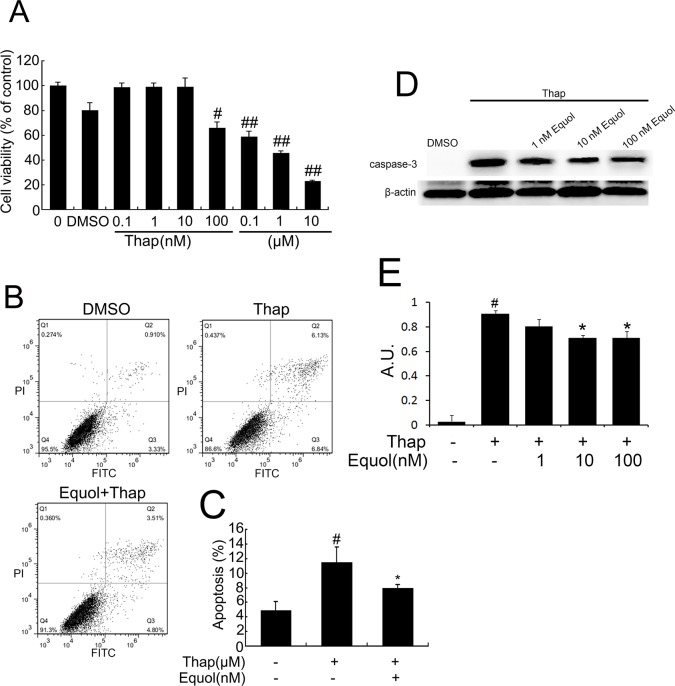
Equol treatment attenuated thapsigargin-induced apoptosis in HUVECs. **(A)** Cells were treated with different concentrations (0, 0.1, 1, 10, 100nM, 0.1, 1, and 10 μM) of thapsigargin (Thap) for 2 h; the medium was then replaced with fresh culture medium, followed by culture for another 22 h. Next, cell viability was measured using a CCK-8 detection kit. **(B)** Thap (1 μM) was added for 2 h and removed before 100 nM equol treatment. Cell apoptosis was then measured by flow cytometry. **(C)** The bar graphs show the quantification of the apoptosis percentage. **(D)** Cells were pretreated with different concentrations (1, 10, and 100 nM) of equol for 24 h followed by treatment with Thap (1 μM) for an additional 2 h, then caspase-3 was determined by western blot. **(E)** The bar graphs show the quantification of the caspase-3 proteins. Values are expressed as means ± SD. n = 3, ^#^*p* < 0.01, ^##^*p* < 0.001 versus the control group; *p < 0.05, **p < 0.01 versus the Thap-treated group.

Equol (1, 10, and 100 nM) treatment significantly attenuated the *t*-BHP-induced decrease in cell viability (Fig 2B). Flow cytometry assessment also showed that equol (100 nM) pretreatment markedly reduced *t*-BHP (Fig 2C and 2D) or thapsigargin (Fig 3B and 3C)-induced cell apoptosis. Simultaneously, equol pretreatment (1, 10, and 100 nM) decrease *t*-BHP (Fig 2E and 2F) or thapsigargin (Fig 3D and 3E)-induced caspase-3 activation.Additionally, equol pretreatment (1, 10, and 100 nM) dose dependently attenuated the increase in ROS levels induced by *t*-BHP (Fig 2G).

### Equol attenuates ER stress both in vitro and in vivo

**Fig 4:**
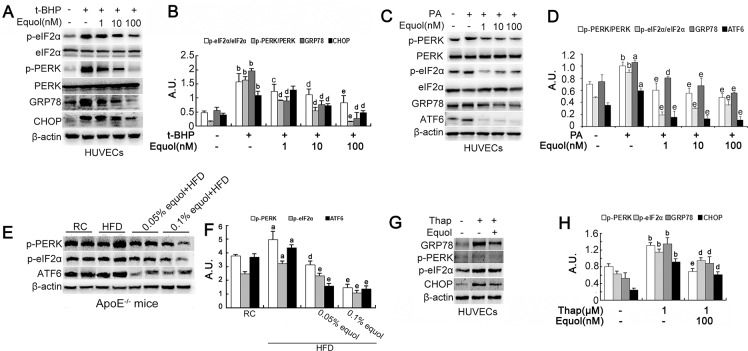
Equol regulated ER stress in vitro and in vivo. **(A)** HUVECs were pretreated with different concentrations (1, 10 and 100 nM) of equol for 24 h, followed by incubation with *t*-BHP (50 μM) for another 6 h. p-PERK, PERK, p-eIF2α, eIF2α, GRP78 and CHOP were detected by western blotting. **(C)** HUVECs were pretreated with different concentrations (1, 10 and 100 nM) of equol for 24 h, followed by incubation with palmitate (PA) (500 μM) for another 24 h. The protein expression of p-PERK, PERK, p-eIF2α, eIF2α, GRP78 and ATF6 was analyzed by western blotting. **(E)** Protein was obtained from the thoracic and abdominal aorta of apoE-/-mice with or without equol treatment, and the expression of p-eIF2α, P-PERK GRP78 and ATF6 was detected by western blotting. **(G)** HUVECs were incubated with Thap (1 μM) for 2 h and removed before equol (100 nM) treatment for another 24 h. The indicated proteins, including p-eIF2α, p-PERK GRP78 and CHOP, were detected by western blotting. **(B)(D)(F)(H)** The bar graphs show the quantification of the indicated proteins. Values are presented as means ± SD. n = 3, ^a^*p* < 0.01, ^b^*p* < 0.001 versus the control group; ^c^*p* < 0.05, ^d^*p* < 0.01, ^e^*p* < 0.001, versus the *t*-BHP-treated group, PA-treated group, HFD or Thap-treated group.

*t*-BHP is a prototypical organic oxidant and has been used extensively to study oxidant-induced cell apoptosis. *t*-BHP-induced ROS accumulation in HUVECs showed partial colocalization with the ER, and previous investigations have confirmed that *t*-BHP could induce ER stress and apoptosis subsequently[17]. Equol (1, 10, and 100 nM) pretreatment effectively inhibited *t*-BHP-induced PERK and eIF2α phosphorylation and GRP78 and CHOP upregulation in a dose-dependent manner (Fig 4A and 4B), which are postulated to attenuate global protein translation, leading to the inhibition of ER stress. Additionally, equol markedly attenuated PA-induced upregulation of p-PERK, p-eIF2α, GRP78 and ATF6 (Fig 4C and 4D). Moreover, equol ameliorated the ER stress inducer thapsigargin-induced elevation of p-PERK, p-eIF2α, GRP78 and CHOP expression (Fig 4G and 4H). These results suggested that equol effectively attenuates ER stress in HUVECs.

In addition, equol intervention, particularly at a high dose (0.1% equol), notably downregulated the levels of p-PERK, p-eIF2α, ATF6 and GRP78 in thoracoabdominal aorta of apoE-/-mice compared with mice fed with HFD (Fig 3E and 3F). These results suggested that equol supplementation mitigated ER stress in apoE-/- mice.

### The Nrf2 signaling pathway plays a role in equol-mediated attenuation of ER stress

**Fig 5:**
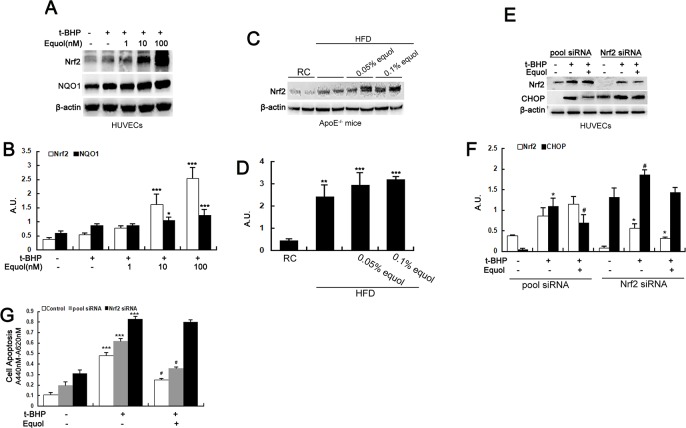
Equol attenuated ER stress by activating Nrf2 in vitro and in vivo. **(A)** HUVECs were incubated with different concentrations (1, 10 and 100 nM) of equol for 24 h before treatment with *t*-BHP (50 μM) for another 6 h. The expression of Nrf2 and NQO1 was detected by western blotting. **(C)** Total tissue lysates from the thoracic and abdominal aorta of apoE-/- mice with or without equol treatment were immunoblotted with anti-Nrf2 and anti-β-actin antibodies. **(E)** Cells were transfected with Nrf2 siRNA for 5~6 h; the medium was then replaced with fresh culture medium, followed by incubation for another 24 h. Thereafter, the cells were treated with equol (100 nM) for 24 h and then were incubated with *t*-BHP (50 μM) for an additional 6 h. The cells were collected and lysed, and western blot analysis was performed. **(B)(D)(F)** The bar charts show the quantification of the indicated proteins. **(G)** HUVECs were transfected with Nrf2 siRNA and treated as described in (C), and cell apoptosis was assayed using the Cell Death Detection ELISA^plus^ Kit. Values are presented as means ± SD. n = 3, *p < 0.05, **p < 0.01, ***p < 0.001 versus the control group, RC group or siRNA treated with no drug group; ^#^*p* < 0.01, ^##^*p* < 0.001 versus the *t*-BHP-treated group and siRNA plus *t*-BHP treated group.

The PERK-Nrf2 signaling pathway plays an important role in the regulation of ER stress. Previous research has identified Nrf2 as a PERK substrate and as being activated during the UPR[18]. Simultaneously, Nrf2 signaling has a cytoprotective role in the response to ER stress[7]. Therefore, the possible role of the Nrf2-mediated signaling pathway in equol-mediated ER stress amelioration was further investigated. Equol treatment (1, 10 and 100 nM) dose-dependently upregulated the expression of Nrf2 and its downstream target gene quinone oxidoreductase 1 (NQO1) in stressed HUVECs (Fig 5A and 5B). A similar tendency was also observed in thoracoabdominal aorta tissues from apoE-/-mice (Fig 5C and 5D). These results indicated that equol activates the Nrf2 signaling pathway both in vitro and in vivo.

The correlation between the activation of Nrf2 signaling and ER stress regulation by equol was further evaluated using Nrf2 siRNA. As shown in Fig 5E–5G, inhibition of Nrf2 observably abrogated the equol-mediated reduction of CHOP upregulation and cell apoptosis in HUVECs under oxidative stress. Taken together, these results imply that the activation of the Nrf2 signaling pathway plays a role in the protective effect of equol against ER stress in HUVECs.

## Discussion

The present study showed that apoE-/-mice fed a HFD containing equol had reduced atherosclerotic lesions compared with mice fed only the HFD. Mechanically, equol feeding reduced ER stress markers in the aortic wall, and equol treatment attenuated ER stress induced by oxidative stress and free fatty acids in HUVECs via Nrf2 induction. These findings suggest that equol ameliorates atherosclerosis development by potentially attenuating ER stress through the upregulation of Nrf2.

Prolonged ER stress is involved in the initiation and progression of several metabolic diseases, including type Ⅱ diabetes, atherosclerosis, neurodegeneration, cancer and liver diseases[19, 20]. Recent studies have highlighted the role of ER homeostasis in endothelial cell function. Pathologically chronic ER stress leads to endothelial cell injury and subsequently to apoptosis, resulting in endothelium impairment, which has been considered to be the initial event in several vascular diseases such as coronary artery disease, peripheral artery disease, hypertension, chronic heart failure and renal failure[21–23]. The present study found that in HFD-fed apoE-/-mice, a high ER stress level was observed in the artery wall. It has been confirmed that ER stress is induced in atherosclerosis lesions[24, 25]. A previous study indicated that ER stress accelerated atherosclerosis through promoting macrophage-derived foam cell formation[26], inducing apoptosis of lesion-resident macrophages[27], accelerating smooth muscle cell proliferation[28], and stimulating the secretion of inflammatory cytokines, adhesion and apoptosis in endothelial cells[29, 30]. These observations are in agreement with the results of the present study. In vitro, we found that an elevated level of ER stress and apoptosis was induced by ROS or palmitate in HUVECs. Endothelial cells plays a pivotal role in maintaining vascular homeostasis, which may interfere with many pathological conditions, including hyperglycemia, insulin resistance, shear stress, homocysteinemia, hyperlipidemia and oxidative stress, resulting in endothelial dysfunction through the activation of ER stress[31]. Saturated fatty acids (SFAs) such as palmitate activate inflammatory pathways and ER stress in different sources of cells, including macrophages[32], cardiac cells[33], pancreatic β cells[34], hepatic cells[35], neurons and endothelial cells[36]. Here, we found that in HUVECs, palmitate induced p-PERK, p-eIF2α, GRP78 and ATF6 expression. Similarly, lipotoxicity, including the induction of ER stress and cell apoptosis exerted by PA, was also observed in mouse microvascular endothelial cells, isolated mouse cardiomyocytes and primary human coronary smooth muscle cells[36]. Additionally, SFAs induced the inflammatory response in human coronary artery endothelial cells[37]. However, the present study did not involve inflammatory analysis. Additionally, a previous study by our group showed that palmitate did not induce cell apoptosis in HUVECs, L6 cells or hepatic cells, even when the adopted dose reached 500 μM.

Although equol is considered to be cardioprotective[38], direct evidence of its antiatherosclerosis effect is limited. The present study demonstrated that equol intervention inhibited the development of atherosclerosis plaques in HFD-fed apoE-/- mice. The only similar report was conducted with daidzein in the same animal model by Nagarajan et al. who found that atherosclerotic lesions were reduced in the aortic sinus and descending aorta in mice fed with soy protein isolate with or without phytochemicals (genistein, daidzein) compared with casein-fed mice[39]. However, the plasma lipid profiles did not differ, and they did not analyze the metabolic equol level in urine or plasma. The present study identified that equol treatment reduced the levels of total cholesterol, triglycerides and LDL-cholesterol and simultaneously increased the HDL-cholesterol level compared with the regular chow-fed group. Eyster et al. demonstrated that equol regulated 10 genes implicated in the presence of atherosclerosis plaques in female cynomolgus monkey iliac arteries, suggesting the potential protective effects of equol in arteries[40]. However, in this study, no significant differences between equol treatment and vehicle groups in atherosclerotic lesions and the plasma lipid profile were identified[40]. The difference in the plasma lipid profile results may be attributed to the different in vivo models. Another study on the influence of equol in adult male rats demonstrated that equol caused a reduction in the plasma levels of total cholesterol, HDL- and LDL-cholesterol and triglycerides[41], a finding that was consistent with our observations.

Equol was found to induce apoptosis in certain cancer cells, including human hepatocellular carcinoma SMMC-7721 cells[42]and MCF-7 breast cancer cells[43]. Its proapoptotic effect was attributed to equol inducing ER stress and inflammatory response stress. However, the present study demonstrated that equol alleviated ER stress induced by palmitate or ROS in HUVECs and protected cells from death induced by thapsigargin, a specific ER stress activator. There is no evidence concerning the function of equol in ER stress in normal cells. Nevertheless, phytochemicals including phytoestrogens such as genistein and daizein often exert cytotoxicity in tumor cells (high/pharmacological dose) and cytoprotection (low/physiological dose) in normal somatic cells. Park et al. demonstrated that treatment of cells with soy isoflavone, genistein and daidzein alleviated homocysteine-mediated neurotoxicity through activation of AMPK against ER stress in SH-SY5Y cells[44, 45]. Kim et al. found that flavonoids including genistein and daidzein down-regulated GRP78 but activated transcription factor 6α (ATF6α), X-box binding protein 1 (XBP1), inositol-requiring protein-1 (IRE1), p-eIF2α and C/EBP-homologous protein, linking their protection to ischemia/reperfusion-induced cardiac damage[46].

Nuclear factor-E2-related factor (Nrf2) belongs to a small family of transcription factors containing a unique basic-leucine-zipper (bZIP) motif, the cap-n-collar (CNC) family[47, 48]. Domain analysis has shown that Nrf2-erythroid cell-derived protein has a CNC homology 1 (Neh1) domain that allows Nrf2 to bind to the antioxidant-response element (ARE) sequence, which controls the transcription of several cellular reducing equivalents such as glutathione (GSH) and NADPH, drug-metabolizing enzymes, transporters and the proteasome[49]. Additionally, the Neh2 domain acts as a negative regulator by binding to the Nrf2 inhibitor Keap1[50]. Under basal conditions, Nrf2 is rapidly degraded through ubiquitination by the cytosolic Keap1/Cul3/Rbx1 complex. However, under stressed conditions such as exposure to electrophiles, ROS and reactive nitrogen species, the ability of ligase to ubiquitinate Nrf2 is inhibited, allowing Nrf2 to translocate to the nucleus and initiate the antioxidant response[48, 50]. The Nrf2-Keap1-ARE signaling pathway is the main mediator of cellular adaptation to redox stress[50]. Here, we found that equol treatment activated Nrf2 in HUVECs and that an elevated level of Nrf2 expression was also observed with equol intervention in HFD-fed ApoE-/-mice. The results are consistent with those of our previous study using the EA hy.926 cell line and HUVECs[51]. In addition, the present study indicated that Nrf2 siRNA interference abolished equol’s attenuation of ER stress induced by ROS or palmitate in endothelial cells. Nrf2 is the most important regulator of cellular resistance to oxidants[52]. Oxidative stress is counterbalanced by complex antioxidant defense systems regulated by a series of multiple pathways, including the UPR, to ensure that the response to oxidants is adequate[7]. Additionally, Nrf2 signaling is required for survival during the UPR. Nrf2-/- cells are sensitive to various ER stress-inducing agents, and Nrf2 overexpression enhances cell survival during the UPR[53, 54]. Therefore, our study suggested that the Nrf2 signaling pathway plays a significant role in equol’s inhibition of ER stress in endothelial cells. Additionally, equol’s ability to activate Nrf2 protected HUVECs from cell injury induced by ROS[51]. However, it is noteworthy that the activation of Nrf2 was considered to be PERK dependent[53]. We found that equol upregulated Nrf2 but downregulated p-PERK and CHOP. The phenomenon may be attributed to other regulators such as ERK1/2 and estrogen receptor β, which exist upstream of Nrf2[51]. In addition, the intervention time, dose of drug and cell type were affected. Future work should emphasize the effect of equol in patients with AS or those with a high risk for cardiovascular disease.
